# Influence of Flow Control Devices on Mixing Phenomena in the Ladle with Top Lance Stirring System—Numerical and Physical Modeling

**DOI:** 10.3390/ma17246130

**Published:** 2024-12-15

**Authors:** Adam Cwudziński

**Affiliations:** Department of Metallurgy and Metals Technology, Faculty of Production Engineering and Materials Technology, Czestochowa University of Technology, Armii Krajowej 19 Ave, 42-201 Czestochowa, Poland; adam.cwudzinski@pcz.pl; Tel.: +48-3432-507-79

**Keywords:** ladle, top lance, flow control devices, mixing phenomena, numerical simulations, water modeling

## Abstract

In this paper, the influence of the structure of the bottom of the ladle with ceramic dam or set of dams on the mixing process was assessed, determining the mixing time required to achieve the level of 95% chemical homogenization. The 0.1 scale water model was used for the physical experiments. The numerical simulations were carried out in the Ansys-Fluent 12.1 software for a 1:1 scale ladle and the behavior of hot metal—nitrogen system. The research focused on three issues, i.e., the influence of the flow rate of technical gas, the influence of the position of the top injection lance, and the influence of the type of dam mounted in the ladle bottom. Finally, the use of a semi-circle dam or set of dams in the ladle bottom together with the top lance being set to a lower depth resulted in a significant reduction in the total mixing time of the liquid metal by 42% and 50%, respectively, without increasing the nitrogen flow rate.

## 1. Introduction

For more than 30 years, numerical methods and physical modeling using the model medium of water have enabled the successful development of treatment metallurgy technologies in the iron and steel area [1,2,3,4]. Hydrodynamics in the ladle is limited not only by gas flow rate but height to diameter ladle ratio [1]. From fundamental works, Mazumdar and Guthrie and Irons et al. clearly point out that gas plume dynamics is balanced by buoyancy and inertial forces [2,3]. Moreover, it is important remember that selection model scale determined methodology of experiments [4].

The hot metal or steel treatment processing comprises processes carried out in batch-type reaction vessels, in which the stirring energy is introduced into the system from the outside. The mixing power depends on the mixing method. Liquid metal can be mixed in the vessel by mechanical or electromagnetic stirrers, or by using technical gases. Technical gases such as nitrogen or argon can be fed into the system by means of gas-permeable plugs placed in the bottom or lances immersed in metal from the top. 

In the mechanical stirring case, the basic criteria for effective mixing are the stirrer immersion depth, the stirrer rotation speed and the type of head limited by the impeller shape and size, and the angle of their position relative to the free metal surface [5,6,7,8,9]. Moreover, by using mathematical models, Wang et al. and Li et al. simultaneously simulated the hydrodynamics and desulphurization rate [6] or the abrasion process [7]. Zhao and his colleagues concluded that the effective sulfur removal from hot metal is strongly influenced by the increase in the rotational speed of the stirrer, while slightly by the impact of its immersion depth in metal. The author showed a close relationship between the sulfur removal from hot metal and the increase in kinetic energy dissipation of turbulence energy caused by the increase in the impeller rotation speed [10]. Nakasuga, on the other hand, improved the efficiency of removing silicon from hot metal using a mechanical stirrer instead of mixing the Ar bath using a lance [11]. In addition, with regard to the mixing time needed to obtain 95% of the chemical homogenization level, Alam and Mazumdar showed that with the same values of energy entered into the system expressed in W/kg, the use of a mechanical stirrer instead of technical gas introduced into the system through a plug located in the bottom of the vessel reduces the above mentioned time [12]. What is more, Alam and Mazumdar showed that intensifying the mixing process could also be successfully achieved by using an electromagnetic stirrer for the mixing process [13]. The efficiency of the metal bath mixing process is also determined by the direction of stirrer rotation and the dynamically changing rotation speed during the process, as well as the additional injection of technical gas by the nozzle or several nozzles in the shaft [14,15]. The mixing process refers not only to the volume of the liquid metal but above all to the interaction of phases coexisting with the metal, i.e., the gas and slag phases of their disintegration and dispersion in the metallic phase, which affects the metallurgical reactions rate between the substrates [14,16]. 

For many years now, the methods of liquid metal electromagnetic mixing in the ladle have been intensively developed [17,18]. The effectiveness of electromagnetic mixing is determined by the type of stirrer used, i.e., the orientation of the coils in relation to the ladle, as well as combined mixing taking into account both the electromagnetic mixing method and the inert gas [13,19]. The combined method not only reduces the liquid metal mixing time as well as intensifies the removal of non-metallic inclusions from it [19]. Among the methods currently used for mixing metal baths, mixing with technical gas is most often used in industrial conditions [20,21,22,23]. 

The effectiveness of the mixing process by means of a gas-permeable plug or a nozzle placed in the ladle bottom is determined by the distance of the fitting relative to the radius describing the ladle bottom. Dai and Niu showed in their work that the non-centric position of the plug effectively reduces the mixing time [24,25]. An additional effect on the mixing process is obtained with two gas-permeable plugs and their different mutual position relative to the main axis of the ladle and the variable gas flow rate in each of them [26,27]. In addition, the increase in the gas flow rate through the plug linearly reduces the mixing time required to achieve the level of 95% chemical homogenization [25]. Li and his colleagues also confirmed the effect of a gas-permeable plug construction on the mixing process, showing that the mixing time is dependent on the forming gas plume in the liquid metal volume [28]. Tan and his colleagues proposed that the flow of gas through the plug should change in pulses as a time function, which further reduces the mixing time [29]. Duan also pointed out in his work that the liquid metal temperature would have an impact on the mixing process, showing that with a certain position of the plug and the argon flow rate, the increase in the degree of liquid metal overheating would reduce the time needed to achieve the required homogenization [30]. In the treatment processes, lances through which the technical gas is blown and the mixing process is stimulated are equally effective. In the case of injection gases via top lances, the following issues are important: the lances number, the lances position, the head design, and the technical gas flow rate [31,32,33,34]. 

From the presented excellent previous work, the mixing power increases most often linearly with greater energy transfer to the system i.e., larger technical gases flow rate, intensive rotor speed or strong electromagnetic stirring, which of course need more electrical energy input. Therefore, to maintain a certain level of mixing stimulant consumption, this paper proposes using a ladle equipped with a flow control device. To present the mixing methods, this paper also proposes that the working volume of the ladle should be modified by using flow control devices in order to optimize the hydrodynamic structure. The additional influence of the ladle’s internal geometry represents a new point in the development of pre- or secondary metallurgy and the intensification of the mixing process. 

Modifying the hydrodynamic structure is a common issue in the flow reactor, which is the tundish. In the tundish, various types of flow control devices in the form of weirs, dams, subflux flow controllers, or gas-permeable barriers are installed. In the tundishes, flow control devices limit the share of stagnant flow and help non-metallic inclusions (NMIs) float to the slag phase [35,36,37,38,39,40,41,42]. Fang et al. showed that over 50% of 10 µm NMIs can be effectively removed from liquid steel depending on how the flow control devices in the tundish are used [36]. Meanwhile, Wang et al. after numerical simulations equipped the industry tundish with proper flow control devices to significantly decrease the NMIs in the billets [39]. Mainly, the flow control devices limit stagnant flow in the tundish. Yue et al. obtained 30% less volume of stagnant flow in the liquid steel bulk by using subflux flow controller and dams [41]. In this paper, the influence of the ladle bottom structure with ceramic dams on the mixing process was assessed, determining the mixing time required to achieve the level of 95% chemical homogenization. 

## 2. Ladle Description 

**Figure 1:**
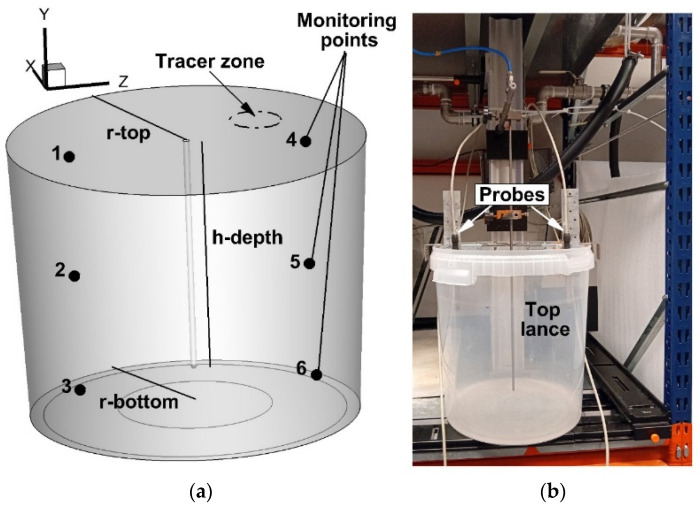
Ladle sketch: (**a**) full scale ladle numerical model, (**b**) 0.1 scale ladle physical model.

**Figure 2:**
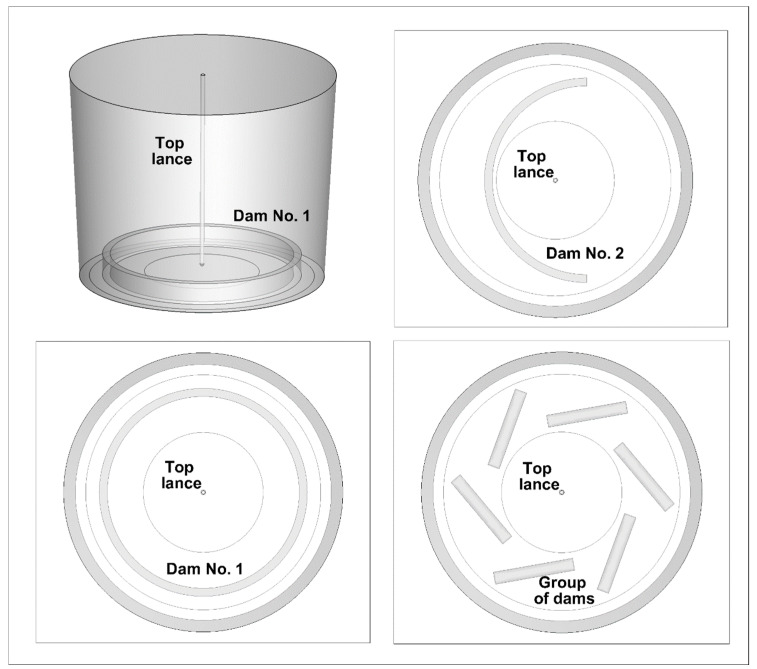
Ladle sketch with locations of considered flow control devices.

The tested object was a ladle designed for hot metal treatment (Figure 1a). The rated capacity of the ladle is 170,000 Mg. The height of the vessel is 3.5 m, while the bottom radius is 1.6 m. The free metal surface radius is 1.745 m, and the height of the metal column is 2.75 m. In the ladle’s central part, there is an injection lance with an internal diameter of 0.04 m. In Figure 1, the feed zone for the tracer is marked with dashed lines. The tracer was injected during physical experiments and numerical simulations. The center coordinates of the tracer feed zone are as follows: Z = 1.2 m, y = 2.75 m, and x = 0.775 m. In the ladle working volume, six measuring points were selected, at which the change in the tracer concentration was recorded. The points were located in the central part of the ladle, that is, the x-coordinate was equal to 0. Points 1 to 3 were 1.3 m away from the lance axis in the opposite direction to the assumed *Z*-axis orientation of the coordinate system under consideration. Points 4 to 6 were offset by the same distance, but according to the *Z*-axis orientation. The mixing process was monitored at three depths of 0.5 m, 1.33 m, and 2.35 m. The measuring points 1 and 4 corresponded to the position of the conductivity probes in the physical model of the ladle (Figure 1b). The ladle physical model was made of plastic in a 0.1 scale (Figure 1b). Three types of dams were installed in the ladle (Figure 2) [43]. The height of all the dams was 0.3 m, while their thickness was 0.1 m. The outer radius of dam No. 1, shaped like a circle, was 1.3 m. Dam number 1 was centered in relation to the longitudinal axis of the injection lance. Half-circle dam No. 2 had the same outer radius, but its position relative to the top injection lance was different. Dam number 2 was moved by 0.4 m according to the x-axis direction. In the ladle working volume, a dams set in the amount of six items was also tested. The length of each dam was the same and was 1 m. The displacement of the dams in relation to the *x*-axis was 30 degrees. While, the dams’ distribution relative to the axis of top injection lance was 60 degrees.

Table 1 presents the research cases considered in the paper. The research was carried out concerning three issues, i.e., the influence of the technical gas flow rate, the influence of the top injection lance position, and the influence of the dam type mounted in the ladle bottom. Two nitrogen flow rates equal to 317 NL/min and 635 NL/min were tested during the research. The transfer of the top injection lance to a distance of ½ and ¼ of the bottom radius counting from the edge of the ladle bottom circle was also tested. In addition, the influence of the top lance that was immersed deeper in a metal bath was verified. Then, with a top lance immersed deeper and a slower flow rate of technical gas, the impact of changing the ladle working volume was verified by its construction with dams.

## 3. Methodology

The basic similarity criterion used in the physical experiments was the Froude’s criterion, in accordance with which were calculated the nitrogen flow rate and the correlation of the liquid hot metal treatment time between the physical model and the numerical model. Froude’s criterion estimates the relation between momentum forces and gravitational forces in the considered system and makes it possible to build a model in the smaller scale. Moreover, water can be used to model hot metal behavior because kinematic viscosity is nearly close for both mediums.

(1) $${\left(\frac{u^{2}}{gL}\right)}_{m}={\left(\frac{u^{2}}{gL}\right)}_{p}$$

where *L*—characteristic length (m), *u*—medium velocity (m/s), *g*—acceleration of gravity (m/s^2^). 

Numerical simulations were carried out in the Ansys-Fluent 12.1 software for a 1:1 scale ladle and hot metal—nitrogen system behavior. In advance, the turbulent nature of system was described via large eddy simulation (LES) approach.

### 3.1. Physical Modeling

The physical model was equipped with the top lance and the flow rate of the air was fully automated so as to ensure repeatability and reaching the required technical gas flow rates for nitrogen flow rate considered in the work. The used methodology for the flow rate of the technical gas and time similarity criteria is valid for an adequate reflect mixing phenomena inside the hot metal bulk created by the gas plume, and it was tested successfully in the author’s previous works [44,45,46,47,48,49].

(2) $$Q_{m}=\lambda^{2.5}Q_{il}$$

(3) $$t_{m}=\sqrt{\lambda }t_{it}$$

where *Q_m_*—volumetric gas flow rate in the physical model, *Q_il_*—volumetric gas flow rate in the industry ladle, *t_m_*—physical trials time, *t_it_*—industry treatment time, *λ*—scale factor.

To standardize the results, the real concentration of the tracer was converted to non-dimensional values, in accordance with Formula (4).

(4) $$F=\frac{C_{t}-C_{0}}{C_{f}-C_{0}}$$

where *C_t_*—temporary tracer concentration, *C*_0_—initial tracer concentration, *C_f_*—final tracer concentration.

The air was injected to the water by the steel made top lance. The gas supply system was powered by a compressor. After establishing the hydrodynamic conditions in the physical model, a 2% NaCl solution of 0.41% of the water weight in the model was introduced into the water at the location tracer in Figure 1a. Conductivity sensors for measurements in dynamic systems are used to measure the specific water conductivity. The air was blown into the model at a flow rate of 1 and 2 NL/min. The recorded changes in the tracer concentration in the water allowed the determination of 95% of the level of chemical homogenization and, subsequently, the determination of the time needed to establish the expected chemical homogenization level. 

### 3.2. Mathematical Model

For an incompressible Newtonian fluid, the continuity and momentum equations take the following form:

(5) $$\frac{\partial {\overset{\text{-}}{u}}_{i}}{\partial {\overset{\text{-}}{x}}_{i}}=0$$

(6) $$\frac{\partial {\overset{\text{-}}{u}}_{i}}{\partial t}+\frac{\partial \left({\overset{\text{-}}{u}}_{i}{\overset{\text{-}}{u}}_{j}\right)}{\partial x_{j}}=-\frac{1}{\rho }\frac{\partial \overset{\text{-}}{p}}{\partial x_{i}}-\frac{\partial \tau_{ij}^{r}}{\partial x_{j}}+\nu \frac{\partial^{2}{\overset{\text{-}}{u}}_{i}}{\partial x_{i}\partial x_{j}}$$

(7) $$\tau_{ij}^{r}=-2\nu_{t}\left(\frac{1}{2}\left(\frac{\partial {\overset{\text{-}}{u}}_{i}}{\partial x_{j}}+\frac{\partial {\overset{\text{-}}{u}}_{j}}{\partial x_{i}}\right)\right)$$

where *u*—velocity, *p*—pressure, *ρ*—density, *ν_t_*—eddy kinematic viscosity, *τ*—stress tensor, *t*—time.

The subgrid-scale model in the LES approaching used in the numerical simulations was the kinetic energy transport model, where kinetic energy and eddy kinematic viscosity are presented by following formulas:

(8) $$k=\frac{1}{2}\left(\bar{uu}-\bar{u}\bar{u}\right)$$

(9) $$\nu_{t}=k^{0.5}CV^{0.33}$$

where *C*—constant, *V*—volume of the computational cell, *k*—kinetic energy.

Gas phase injections were described using the Discrete Phase Model (DPM) with stochastic bubbles behavior in the metal volume (Discrete Random Walk), described in detail in this work [47]. The numerical simulation took into account the gas expansion. The numeric grid was made of approx. 0.55 million tetrahedral elements, according with methodology presented in the work [22]. More detailed data on the numerical model and physico-chemical properties of hot metal and nitrogen were described in the author’s previous work [50].

## 4. Results and Discussions

### 4.1. Model Validation—Mixing Time

**Figure 3:**
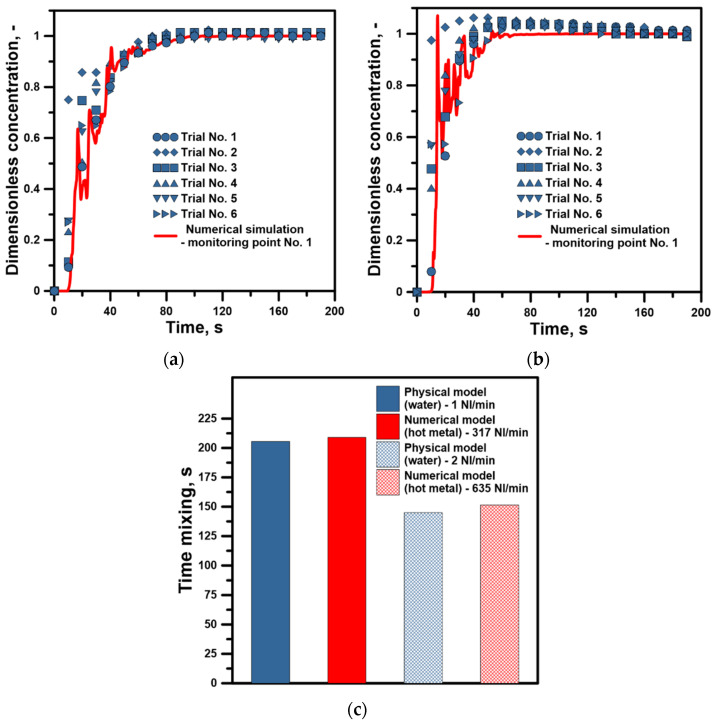
Numerical model validation: (**a**) mixing curve for N_2_ flow rate: 317 NL/min, (**b**) mixing curve for N_2_ flow rate: 635 NL/min, (**c**) time mixing for both considered flow rates.

The water model was used to validate the numerical model of blowing hot metal with nitrogen. Due to the fact that the water model was a fully isothermal model with room temperature and a very small height of the water column, the gas bubbles expansion process during experiments could be disregarded. Therefore, to obtain comparable hydrodynamic conditions, the numerical model did not take into account at this stage the process of gas bubbles expansion. Figure 3 presents the behavior of the tracer concentration in the water and calculated mixing time. The numerical model used with the description of the system turbulence using the LES method correlates very well with the results obtained on the water model. Although there are temporary deviations in the initial stage of the tracer’s dissolution, high-temperature systems with such high mixing dynamics of the gas stream forming local hydrodynamic structures often evolve in a way difficult to describe even by advanced numerical algorithms. However, the average mixing time of two conductivity probes calculated from the physical model was 205.5 s, which is less than the mixing time calculated from the numerical model by 3.5 s. In the case of a 100% increase in the technical gas flow rate, the average mixing time from two measuring areas obtained in the water model was 145 s and was lower than that obtained from computer calculations by 6.5 s. The obtained results prove that the numerical model is very useful for describing the macromixing process in the batch reactor, which is the analyzed 170 ton ladle. 

### 4.2. Numerical Simulation—Influence of Nitrogen Flow Rate, Lance Position and Flow Control Devices on Mixing Time

**Figure 4:**
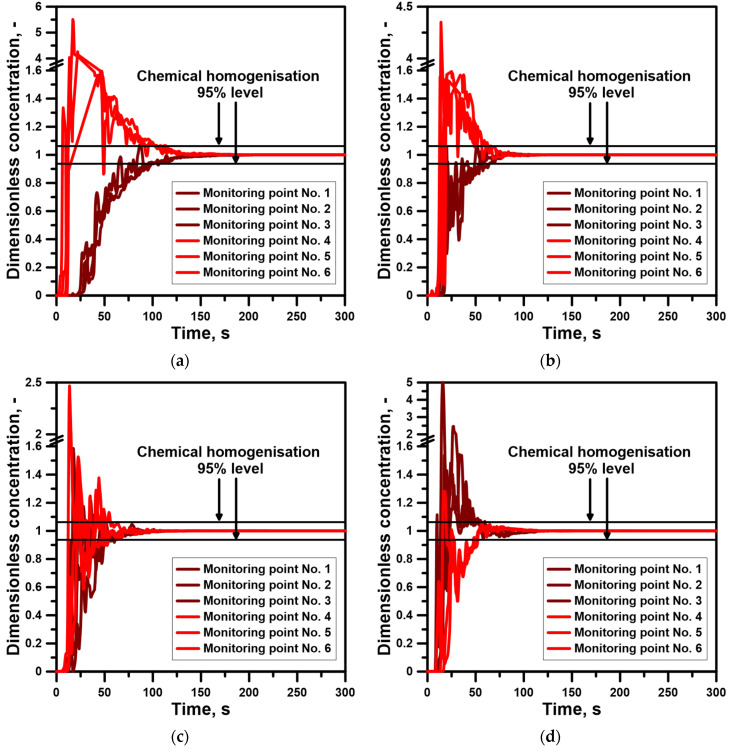
Mixing curves: (**a**) case No. 1 of hot metal stirring, (**b**) case No. 2 of hot metal stirring, (**c**) case No. 7 of hot metal stirring, (**d**) case No. 8 of hot metal stirring.

The verified numerical model was used to simulate the macromixing process of the metal bath in the various conditions of the technical gas injection, lance position and ladle bottom structure. At this research stage, the nitrogen blowing process into the hot metal took into account the process of gas bubble expansion. The expansion effect is visible in the distribution of mixing curves monitored at measuring points 1–6 for the variant with a centrally located top injection lance and a nitrogen flow rate of 317 NL/min and 635 NL/min. Figure 4a,b show a marked change in the curves shape and an intense peak decay, resulting in the final flatness of the curves and their tails being placed in an area of 95% chemical homogenization. The intensive flatness of the curves is further intensified by the dams installed in the ladle bottom (Figure 4c,d). However, if a dams set is installed in the ladle bottom, the nature of the curves at measuring points 1 to 3 changes completely. The curves recorded at these points do not increase in the direction of the expected chemical homogenization zone and fall to the above mentioned zone.

**Figure 5:**
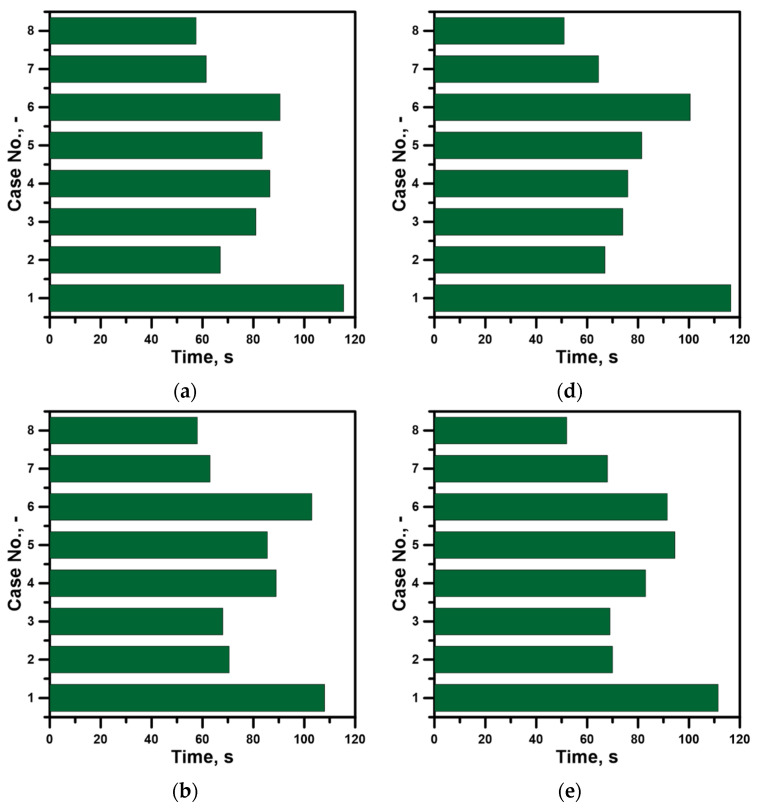

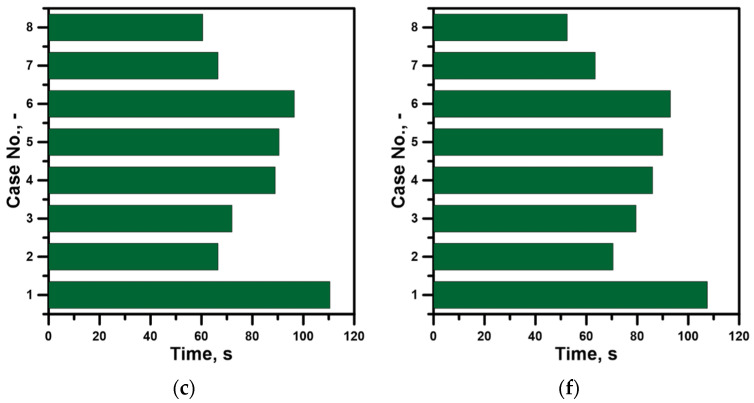
Local mixing time: (**a**) measurement point No. 1, (**b**) measurement point No. 2, (**c**) measurement point No. 3, (**d**) measurement point No. 4, (**e**) measurement point No. 5, (**f**) measurement point No. 6.

In the case of treatment processes, the optimal mixing time is sought, and there is no uniform distribution in the metal volume. Therefore, the procedure for determining the mixing time for the object to be examined should take into account the different ladle working volume zones. In a ladle with a central top lance and a nitrogen flow rate of 317 NL/min, the local mixing time ranged from 116.5 to 107.5 s (Figure 5a–f). One hundred percent increase in nitrogen flow reduces the local mixing time to a range from 66.5 to 70.5 s. At the same time, it is confirmed that intensive liquid metal mixing not only reduces the mixing time but also improves the process by creating its homogeneity. It is obvious that the increase in the technical gas flow rate will intensify the mixing phenomenon, contributing to a reduction in the mixing time. However, it is interesting to obtain a reduction in the mixing time without increasing the consumption of technical gas. By changing the lance position to non-centric according to literature data, the mixing process was expected to improve. When the lance was placed in the ½ position of the bottom radius, the local mixing time oscillated between 68 and 81 s. At the same time, the distribution at individual liquid metal depths was quite heterogeneous. If the lance is moved further toward the side wall, the local mixing time increases slightly locally to 89 s. In the lance positioning area, the immersion depth has been increased by 0.3 m, with a centric position. As a result, the mixing time was reduced by extending the formation path of the gas plume by the rising gas bubbles, creating conditions for their more effective impact on the metal bath. The maximum local mixing time for this stirring case was 94.5 s.

Deeper immersion of the top injection lance was also used for the variant of the ladle with dams placed in the bottom. For the ladle with dam No. 1, the local mixing time at the two monitoring points exceeded 100 s. This variant is also characterized by a considerable asymmetry of mixing times with the top injection lance, except for the zone closest to the ladle bottom, where the difference in local mixing time between point 3 and 6 was 3.5 s. The ladle with a semi-circular dam located in the bottom of the ladle asymmetrically in relation to the top injection lance has a range of local mixing times from 61.5 to 68 s. In addition, the differences in mixing times between the individual pairs of points located at the metal bath depth levels differed by a maximum of 5 s. If a dams set is used, the differences between the individual pairs of measuring points are a maximum of 8 s. However, the minimum local mixing time drops to 51 s.

**Figure 6:**
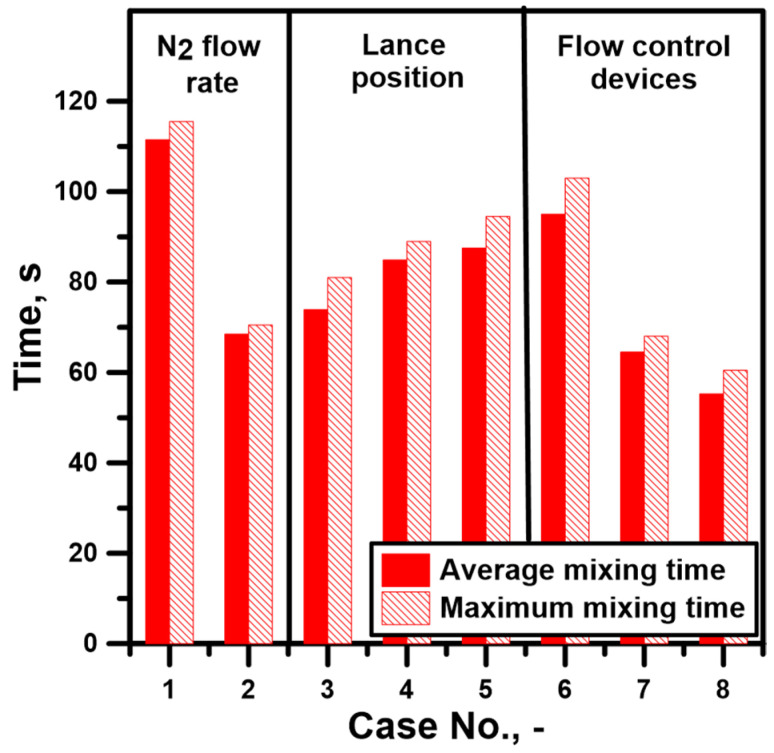
Total mixing time for considered hot metal stirring conditions.

Figure 6 shows the results of the total mixing time, taking into account the maximum local mixing times that limit the process performance. When the flow rate increases from 317 NL/min to 635 NL/min, the total mixing time decreases by 43 s. With the eccentric position of the injection lance in the ½ r position, the mixing time is reduced by 37.6 s at a nitrogen rate of 317 NL/min. Not least, when the process is affected by a further lance shift, at the ¼ r position, the mixing time reduction is 26.6 s. However, bearing in mind that positioning the lance relative to the ladle bottom radius is not always possible in industrial conditions, it was decided to increase the immersion depth of the top lance in a metal bath, which resulted in a mixing time reduction by 24 s. The weakest effect on the mixing process was obtained by using the number 1 dam in the ladle, eliminating the positive effect of the top lance immersion smoothness and achieving a mixing time reduction by only 16.5 s. On the other hand, the construction of the bottom of the ladle asymmetrically set with dam No. 2 or a group of dams resulted in a reduction in mixing time by 47 and 56 s in succession, without increasing the nitrogen flow rate. Analyzing the differences between the maximum local mixing time and the total mixing time, it was found that increasing the nitrogen flow rate to 635 NL/min reduces the value of this time to 2 s. However, values of 7, 8 s were obtained with the non-centric arrangement of the lance (½ r), its deeper immersion, and the use of dam No. 1. In these variants, the local mixing time will most intensively slow down the mixing process. However, in the most advantageous versions of the considered ladle type, the difference between the above mentioned sometimes reaches values of 3.5 and 5.2 s successively, for the ladle with dam No. 2 and the ladle with the dams set. 

### 4.3. Numerical Simulations—Influence of Nitrogen Flow Rate, Lance Position and Flow Control Devices on Hydrodynamics Inside Hot Metal Bulk

**Figure 7:**
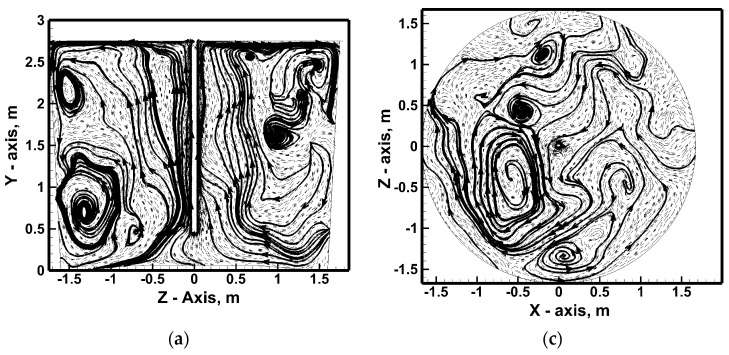

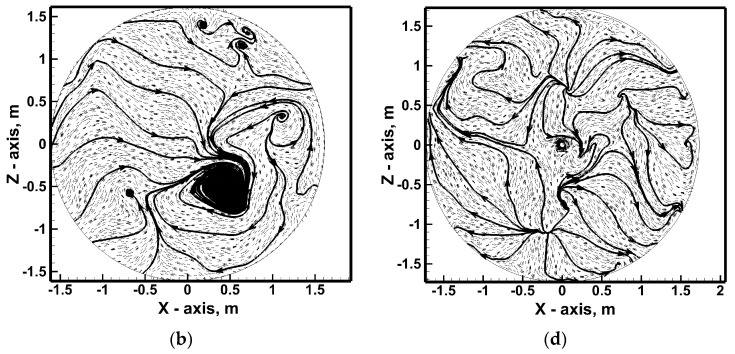
Hydrodynamics inside hot metal bulk (stirring case No. 1): (**a**) vertical central plane, (**b**) horizontal plane at level 0.25 m from ladle bottom, (**c**) horizontal plane at level 1.375 m from ladle bottom, (**d**) horizontal plane at level 2.5 m from ladle bottom.

**Figure 8:**
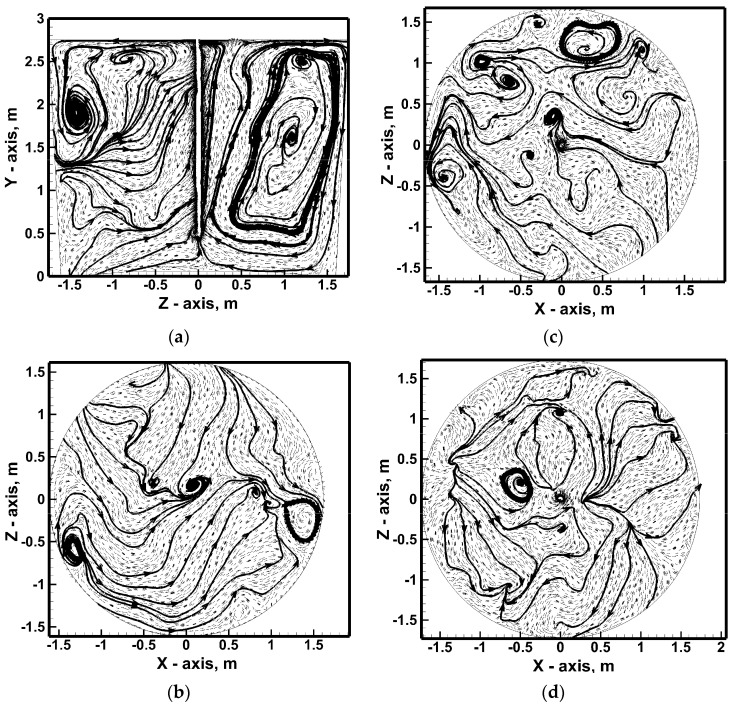
Hydrodynamics inside hot metal bulk (stirring case No. 2): (**a**) vertical central plane, (**b**) horizontal plane at level 0.25 m from ladle bottom, (**c**) horizontal plane at level 1.375 m from ladle bottom, (**d**) horizontal plane at level 2.5 m from ladle bottom.

**Figure 9:**
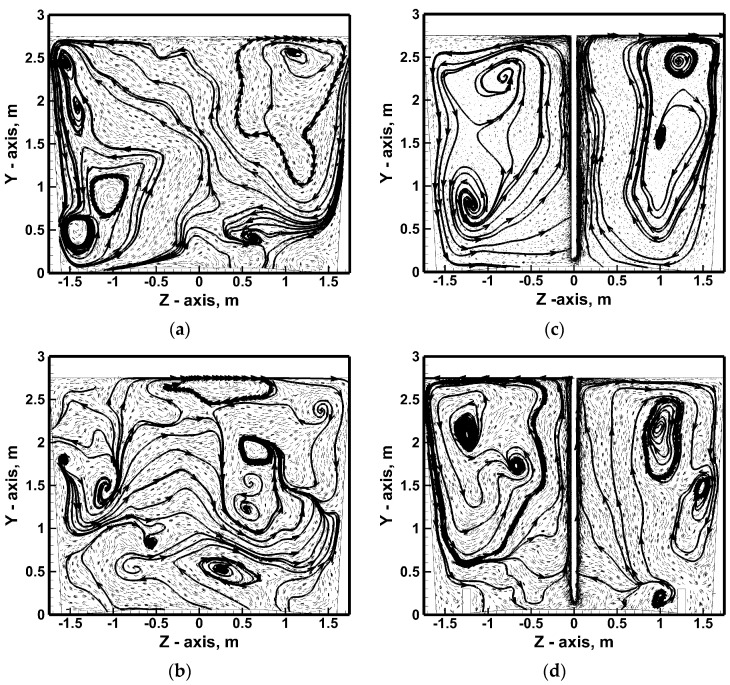
Hydrodynamics inside hot metal bulk—vertical central plane: (**a**) stirring case No. 3, (**b**) stirring case No. 4, (**c**) stirring case No. 5, (**d**) stirring case No. 6.

**Figure 10:**
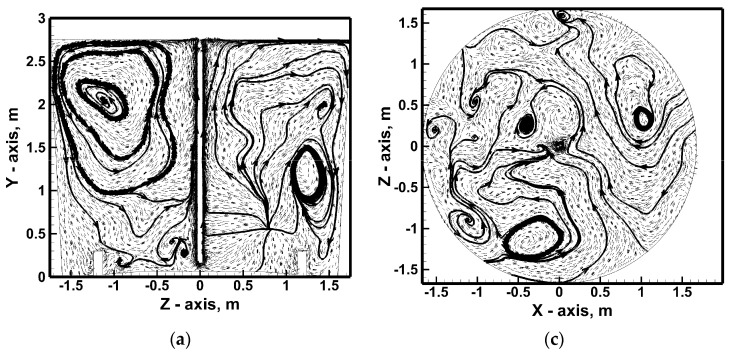

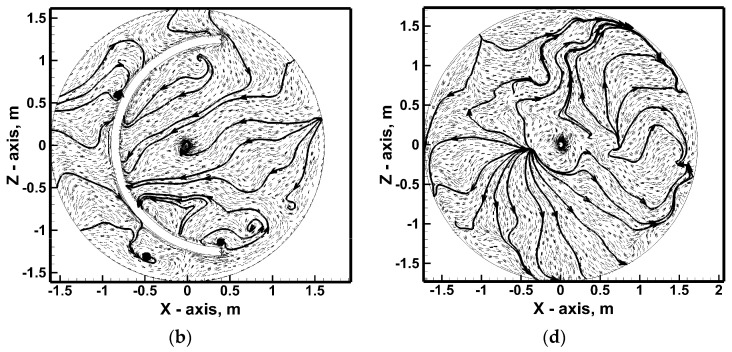
Hydrodynamics inside hot metal bulk (stirring case No. 7): (**a**) vertical central plane, (**b**) horizontal plane at level 0.25 m from ladle bottom, (**c**) horizontal plane at level 1.375 m from ladle bottom, (**d**) horizontal plane at level 2.5 m from ladle bottom.

**Figure 11:**
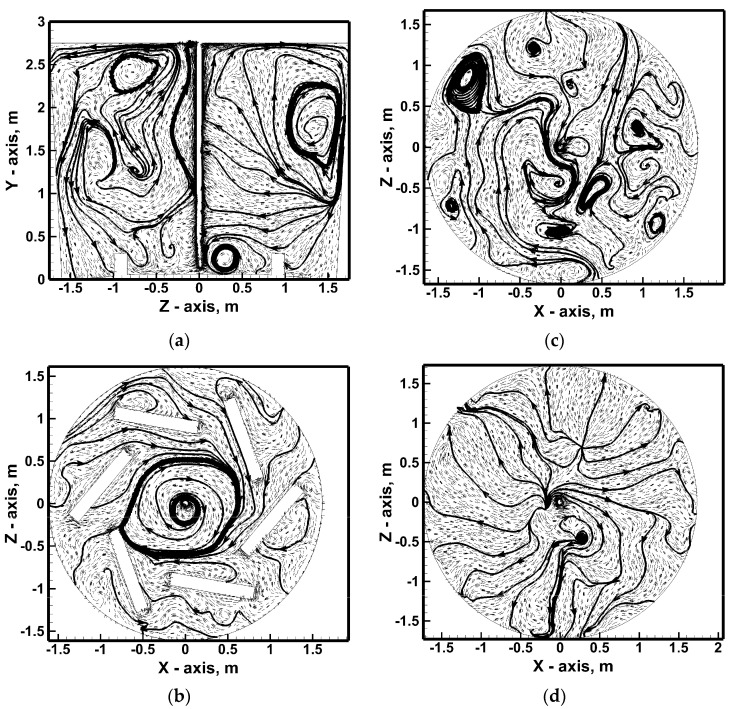
Hydrodynamics inside hot metal bulk (stirring case No. 8): (**a**) vertical central plane, (**b**) horizontal plane at level 0.25 m from ladle bottom, (**c**) horizontal plane at level 1.375 m from ladle bottom, (**d**) horizontal plane at level 2.5 m from ladle bottom.

Figure 7, Figure 8, Figure 9, Figure 10 and Figure 11 show the hydrodynamic structures forming in the ladle during considered stirring conditions. Four planes were selected. The first plane is the plane perpendicular to the bottom of the ladle and located in the reactor central part. In contrast, planes parallel to the ladle bottom are located at three depths of immersion in hot metal, counting from the bottom of the ladle at heights of 0.25 m, 1.375 m, and 2.5 m. Most often, the hydrodynamic structure of the metal streams flow on a plane perpendicular to the ladle bottom at the place of the gas plume interaction is homogeneous in the context of the formation of two distinct vertical liquid metal circulation (a common image when using the RANS model in the numerical model). However, when using the LES approach, a nitrogen flow rate of 317 NL/min is applied simultaneously with the ascending system of liquid metal streams in the top injection lance area. Smaller areas of vertical and horizontal recirculation are additionally formed in the metal volume (Figure 7a).

Also, a 100% increase in nitrogen flow rate does not completely homogenize the hydrodynamic structure, although the number of smaller recirculating areas is reduced (Figure 8a). At the ladle bottom, recirculating the stream’s flow from the ladle side wall toward the center of the reactor, with a hydrodynamic system of 635 NL/min, was more symmetrical in relation to the ladle center (Figure 7b and Figure 8b). In the middle of the height of the metal column on the transverse plane, there is a fairly homogeneous (Figure 7c) and heterogeneous (Figure 8c) distribution of smaller recirculating zones. In the nitrogen flow rate case of 317 NL/min, after reaching the free surface of hot metal, the streams from the lance zone flow toward the edges of the lateral wall along which they then fall toward the ladle bottom (Figure 7d). In the case of an increase in nitrogen flow, the structure of 0.25 m below the free surface of the hot metal is more complex (Figure 8d). On the one hand, the streams flow from the center toward the side walls. On the other hand, some of the streams flow from the side wall toward the center. Figure 9 shows the hot metal streams distribution on the vertical plane for variants including lance positioning. Moving the injection lance by ½ r or ¾ r from the center of the vessel causes deformation of the vertical recirculation fronts, and in the case of lance located close to the ladle side wall, the flow evolves to move horizontally with local micro-areas of horizontal metal circulation (Figure 9a,b). By contrast, increasing the immersion depth of the injection lance effectively homogenizes the vertical metal recirculation structure on both sides of the top injection lance, giving it a systematic direction from the center toward the side wall (Figure 9c). 

Dam No. 1 changes the macro-nature of hot metal circulation in the analyzed vertical plane by activating in the metal volume the local circulation areas of the hot metal streams coexisting with the two macro-areas of upward recirculation at the lance and downward at the ladle side wall (Figure 9d). Two main vertical recirculation zones are maintained in the ladle with dam No. 2, although they are slightly different in shape compared to the case of the metal bath stimulation variant using the top injection lance only (Figure 10). The modification is particularly visible in the zone above the dam; the streams in this part of the vessel flow toward the lance above the dam. By installing the dam, the liquid metal movement in this part of the ladle working volume is modified by creating the effect of the dam flowing through the recirculating streams in a horizontal arrangement (zone up to ¼ of the metal column height). In the middle of the metal volume, the dam initiates more micro-areas of metal circulation. On the other hand, under the hot metal-free surface, a more systematic hydrodynamic structure is observed in the form of two distinct areas of the flow of streams from the ladle center toward the side wall. In the case of a ladle equipped with a dams set, it can be seen on the vertical plane that the hydrodynamic structure is clearly divided into two areas of vertical and horizontal circulation (Figure 11). For approximately up to 1/3 of the liquid metal column height, the hot metal circulates in a horizontal arrangement. This arrangement is particularly pronounced on a plane parallel to the bottom at a height of 0.25 m. In addition, in the middle of the liquid metal column height, there is a significant amount of horizontal micro-circulation quite homogeneously distributed in the liquid metal volume. On the other hand, in the upper part of the hot metal volume, the hydrodynamic structure is homogenized, in which streams flow from the center toward the ladle side wall. 

Table 2 presents the global and local values of the key quantities describing the turbulence system in the hot metal bulk. The average global values are the mean values inside hot metal bulk. The local values of the energy dissipation rate and velocity were read from a point below 0.03 m below the hot metal free surface in the central position of the tracer injection zone. A 100% increase in the nitrogen flow rate occurs at a 38% higher hot metal average velocity in the ladle volume. By contrast, the asymmetric location of the top lance slightly decreased the average of the hot metal velocity, but it did not negatively influence the mixing time. The deeper position of top lance and equipping the ladle with flow control device increased the average velocity maximum at 11%. The more intense agitate of the hot metal causes the formation of the flow vorticity to have a value of 4.1 1/s. A slight increase in the vorticity becomes apparent when the proposed flow control devices are used in the ladle while keeping a lower nitrogen flow rate.

On the other hand, the global dissipation rate changed significantly only when the nitrogen flow rate increased. This view was confirmed by the values of the global turbulent viscosity, e.g., a 30% change was obtained for a 635 NL/min nitrogen flow rate. Rises and falls of global quantities describing the turbulence system did not correlate with the obtained tendency on the mixing time. Therefore, additional analysis in the selected zone of the tracer injection was performed. The hot metal velocity in the tracer injection zone values was the same even when the nitrogen flow rate was equal to 635 NL/min. By contrast, the local velocity decreased 75% for the same 317 NL/min nitrogen flow rate, but after deeper immersion of the top lance and mounting dam No. 1 in the ladle.

Very interesting are the local dissipation rate values in the tracer injection zone when e.g., the 40–60% lower values were obtained in the cases of the ladle with the flow control devices (Table 2). It is noted that the global dissipation rate values for these cases are nearly the same. The results confirm the complexity of the considered system where the proposed FCD effectively modifies the hydrodynamics and creates local conditions for efficiency mass transport phenomena in the hot metal bulk.

## 5. Summary

Based on laboratory investigations, including computer simulations and water model experiments, the effect of the nitrogen flow rate, top lance position, and flow control devices on the mixing process in the considered 170 ton ladle was examined. From the obtained results, it was found that

1. For the considered ladle type, a one hundred percent increase in nitrogen flow rate to 635 NL/min contributes to a 38.5% reduction in total mixing time.
2. The non-centric positioning of the top injection lance or its deeper immersion in a metal bath at a fixed nitrogen flow rate of 317 NL/min reduces the total mixing time by an average of 26%.
3. Deeper immersion of the top lance together with semi-circle dam in the bottom of the considered ladle resulted in a significant reduction in the total mixing time of the metal bath by 42%, with a nitrogen flow rate of 317 NL/min. However, the most advantageous solution for reducing the mixing time is the use of the dams set. In this solution, a 50% reduction in mixing time was achieved.
4. The mixing process with the top injection lance can be effectively intensified by ceramic dams mounted at the bottom of the considered ladle, resulting in the effect of creating homogeneous zones of horizontal liquid metal circulation. Of course, future investigations on the influence of other flow control device configurations, different shape and capacity of the ladles, and the location of tracer injection are obligatory.
5. The considered flow control devices significantly modified the local hydrodynamic hot metal flow structure and gave additional paths for faster mass transport in the metal bulk. This is a starting point of future investigation on the estimate parameters that limit the mass transport phenomenon in the ladles with flow control devices.

## Figures and Tables

**Table 1:** Considered variants of ladle top lance stirring process.

Case No.	Nitrogen Flow Rate, NL/min	Lance Depth Level, m	Lance Position in Relation to Ladle Radius	Type of Flow Control Devices
1	317	2.31	1r	not considered
2	635	2.31	1r	not considered
3	317	2.31	1/2r	not considered
4	317	2.31	1/4r	not considered
5	317	2.61	1r	not considered
6	317	2.61	1r	dam No. 1
7	317	2.61	1r	dam No. 2
8	317	2.61	1r	dams set

**Table 2:** Global and local turbulence system in the hot metal bulk.

Case No.	Average Global Velocity, m/s	Global Vorticity, 1/s	Global Turbulence Kinetic Energy Dissipation Rate, m^2^/s^3^	Global Average Turbulence Viscosity, Pa·s	Local Velocity, m/s	Local Turbulence Kinetic Energy Dissipation Rate, m^2^/s^3^
1	0.26	3.1	0.0013	0.33	1.19	0.0068
2	0.36	4.1	0.0027	0.43	1.19	0.0126
3	0.25	3.0	0.0012	0.32	1.15	0.0073
4	0.23	2.9	0.0015	0.30	0.39	0.0058
5	0.30	3.2	0.0013	0.34	0.74	0.0051
6	0.27	3.3	0.0012	0.32	0.31	0.0043
7	0.26	3.3	0.0013	0.31	0.81	0.0034
8	0.29	3.4	0.0013	0.34	0.94	0.0028

## Data Availability

The data are contained within the article. Raw data that support the findings of this study are available from the Author upon request.
